# Peer education: The effects on knowledge of pregnancy related malaria and preventive practices in women of reproductive age in Edo State, Nigeria

**DOI:** 10.1186/1471-2458-11-610

**Published:** 2011-08-01

**Authors:** Petra F Mens, Pauline FD Scheelbeek, Hind Al Atabbi, Ehijie FO Enato

**Affiliations:** 1 Koninklijk Instituut voor de Tropen (KIT)/Royal Tropical Institute, KIT Biomedical Research, Meibergdreef 39, 1105 AZ Amsterdam, The Netherlands; 2 Department of Clinical Pharmacy & Pharmacy Practice, Faculty of Pharmacy, University of Benin, Benin City 300001, Nigeria

## Abstract

**Background:**

There is limited uptake of measures to prevent malaria by pregnant women in Nigeria which is often related to the lack of knowledge on Malaria in Pregnancy (MIP) and its effects on mother and foetus. This study, explored peer to peer education as a tool in raising knowledge of MIP among women of child bearing age.

**Methods:**

1105 women of child bearing age were interviewed in their households using a structured questionnaire about their knowledge of malaria in general, MIP and use of preventive measures. Thereafter, a peer education campaign was launched to raise the level of knowledge in the community. The interviews were repeated after the campaign and the responses between the pre- and post-intervention were compared.

**Results:**

In the pre-assessment women on average answered 64.8% of the question on malaria and its possibility to prevent malaria correctly. The peer education campaign had a significant impact in raising the level of knowledge among the women; after the campaign the respondents answered on average 73.8% of the questions correctly. Stratified analysis on pre and post assessment scores for malaria in general (68.8 & 72.9%) and MIP (61.7 & 76.3%) showed also significant increase. Uptake of bed nets was reported to be low: 11.6%

**Conclusion:**

Peer education led to a significant increase in knowledge of malaria and its prevention but we could not asses its influence on the use of preventive measures.

## Background

*Plasmodium falciparum *malaria during pregnancy poses a substantial risk to mother and foetus; it leads to an estimated 10,000 maternal anaemia-related deaths in sub-Saharan Africa annually [1]. Furthermore, malaria during pregnancy leads to an increased risk of low birth weight (LBW) in neonates and is responsible for up to 35% of preventable LBW neonates in malaria-endemic areas [2].

In recent years, convincing evidence has shown that preventive methods such as the use of insecticide treated bed nets (ITNs) and intermittent preventive treatment in pregnancy with sulphadoxine-pyrimethamine (IPTp-sp) can greatly reduce the adverse effects of malaria during pregnancy [3,4], and since 1998 this measures has been recommended by the World Health Organization (WHO).

IPTp and ITNs are key components of the National Malaria Control Program of the Nigerian Ministry of Health, and these strategies are expected to reduce the intolerable burden of malaria during pregnancy in the country [5]. The Nigerian government promotes IPTp for pregnant women; however, at the moment, not all states are distributing this intervention free-of-charge when women visit the ANC whilst pregnant. In addition, regular ITN advocacy campaigns are being held including free distribution to pregnant women, often in collaboration with local NGOs. Despite the evidence of the successes of ITNs and IPT-sp, the uptake and coverage in Nigeria is surprisingly low [6-9] and thus reasons for the low uptake and subsequent measures to increase the uptake are being sought.

Several studies have suggested that both adherence to malaria treatment and uptake of malaria prevention activities are linearly associated with knowledge of the adverse health effects of malaria [10,11]. Success rates of any treatment or prevention focused intervention is, thus, likely to be determined by the level of awareness of the risks of malaria and the level of knowledge on available strategies to prevent it.

This hypothesis implicates the need for improved education campaigns to enhance the knowledge of women about malaria and the possible preventive actions they can take. The design and key messages of such awareness campaigns are not easy to determine and depend on the local cultural background and structural possibilities. In many rural areas the possibilities of educational campaigns are limited, because of structural barriers such as poor access to professional health services and limited availability of media such as books, television or radio [12].

Peer education has shown to be an effective tool to improve knowledge in larger groups of people in those areas where possibilities for conventional education methods, such as radio adverts or education through health centers are limited. The method has been successfully used to increase community knowledge on reproductive health, HIV/AIDS prevention, drug abuse, as well as awareness and knowledge in breast cancer and self examination of breasts campaigns [12-15].

Following these successful examples, a peer education campaign was developed in order to increase level of knowledge about adverse health effects of malaria during pregnancy and uptake of preventive practice among women of child bearing age in Edo State, Nigeria. On the average, between 75-80% of pregnant women experience malaria during their pregnancy [9] in the country, and only a limited preventive practices are being performed [16].

This study assesses the effects of a peer education campaign on knowledge of malaria in pregnancy, and uptake of preventive practices in women of child bearing age.

## Methods

### Study area

**Figure 1:**
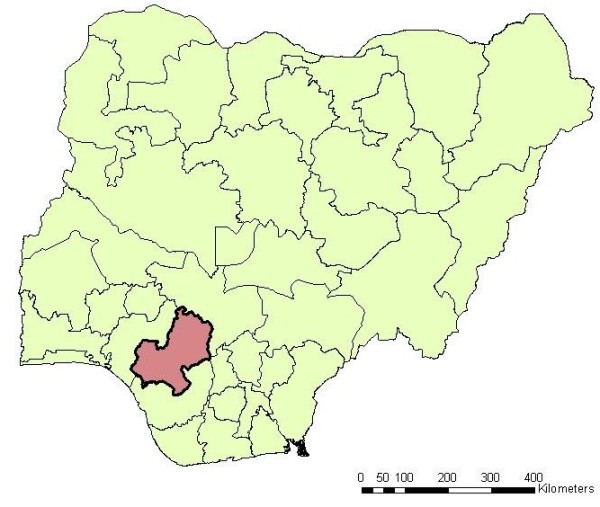
**Location Edo State within Nigeria**.

**Figure 2:**
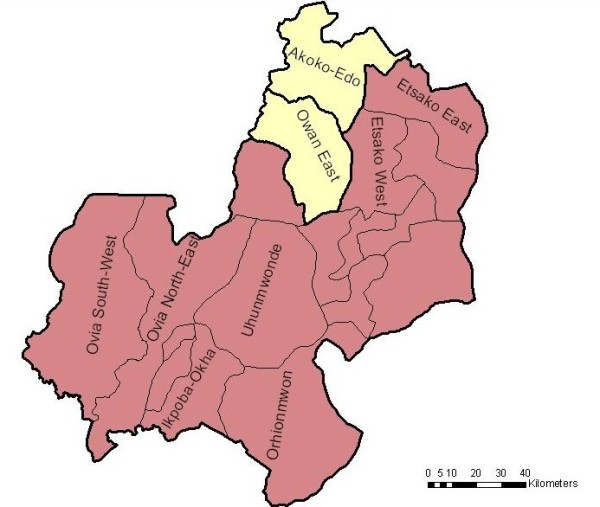
**Location of selected LGAs within Edo State**.

The study was performed in 2009 and carried out in two Local Government Areas (LGAs) in Edo State, Nigeria (Figure 1); Owan-East and Akoko-Edo (Figure 2). In both LGAs, malaria is a major public health problem. Within each of the LGAs three clusters/communities were selected based on urban status and geographical position. These six clusters include 33 Enumeration Areas (EAs), representing 7% of all EAs in the two LGAs. The selected clusters were: Afuze (urban), Ikao, and Olele-Erah (both rural) from Owan-East LGA, and Igarra (urban), Ojah, and Ugboshi-Affe (both rural) in Akoko-Edo LGA. Based on 2006 National Population and census figures, the population in the two LGAs are 154,385 inhabitants for Owan-East and 262,110 inhabitants for Akoko-Edo. Farming is the main occupational activity in the locality, while only a few people serve as civil servants, especially those living in Afuze and Igarra, the administrative headquarters of the two LGAs.

### Study population

In the selected clusters, door-to-door household numbering and listing of household occupants was organised in order to identify all women of child bearing age. Before the intervention took place, consent was asked from eligible women (residents and visitors) to participate in the study. After the peer-education campaign this process was repeated; the same households were visited and the women were approached for participation in the post-intervention assessment.

### Study design

The study was conducted in three phases. In Phase I, a survey of knowledge, attitude and practice (KAP) of the study participants was undertaken by trained staff (interviewers and supervisors). This was followed by Phase II; peer education by trained peer educators through workshops, rallies, and door-to-door campaigns. Finally, in Phase III the post- intervention survey was carried out using the same method and sample frame as in pre-intervention. Phase III took place six months after Phase II.

In total, 30 peer educators selected from the clusters participated in the study. Women were selected based on their active involvement in community-oriented programmes. Field workers who were themselves members of the community assisted in identifying them. The educators were trained during three extensive sessions by experts on malaria and malaria in pregnancy from the University of Benin, Nigeria and the *Roll Back Malaria *Programme of Edo State Ministry of Health. They were given general information on malaria, how is it transmitted, how it can be prevented, etc, with special attention given to the risks of malaria in pregnancy, the need to attend ANC's, the use of SP to prevent malaria during pregnancy. In addition, they were instructed on the importance of and the use of insecticide treated bed nets. Also, tools such as a "ten commandments on malaria in pregnancy list" stating the ten key messages on malaria in pregnancy and a booklet with tips on how to organize peer to peer sessions were given. Immediately after the sessions, a post test was taken from each peer educator, to verify the understanding and level of knowledge of the peer educators. If the level of knowledge was up to standards, the educators were asked to organize peer- to-peer education sessions in their communities. The sessions the peer educators gave consisted of house to house visits and group discussions organized by the peer educators themselves. In addition two rallies were organized, one in each LGA, in which the peer educators also spread their knowledge via discussions and workshops.

### Data collection methods

Questionnaires were used for data collection. The design of the questionnaire was based on review of literature and professional experience of the investigators. Furthermore, questionnaires developed for previous studies done in the locality [9,16] and from the *Malaria Indicator Survey *by the ORC MACRO, and *Guideline for Core Malaria Indicators for population coverage *developed by the Roll Back Malaria [17,18] were adapted for use in this study.

The questionnaire was arranged into 5 sections; (1) demographics, (2) knowledge of both malaria and malaria in pregnancy, (3) uptake of preventive practices during previous pregnancies, (4) current pregnancy status, antenatal care attendance, and history of malaria and (5) bed net ownership and household observation. The questions were "multiple choice" question, with sometimes a single and sometimes multiple answer options.

The questionnaire was piloted on a group of women from Benin City and revisions were made before the survey was conducted. Prior to the survey, the interviewers (n = 12) and supervisors (n = 2) were recruited from the study communities and given an intensive classroom and field-based training as well as a detailed manual of survey guidelines. Two interviewers were allocated to each cluster/community to conduct the survey. Each LGA had one supervisor during the pre- and post-intervention studies.

### Ethical considerations

The study was approved by the Ethical Committee of the University of Benin Teaching Hospital, Benin City, Nigeria. In addition, administrative approvals were sought and obtained from the administrative heads of the respective LGAs.

### Data analysis

Baseline tables were created to summarize demographic characteristics of the study participants.

Level of knowledge and uptake of preventative practice was expressed in scores (percentages) ranging from 0 (very low level of knowledge/no uptake of preventative practices) to 100 (very high level of knowledge/uptake of all available preventative practices). This score was calculated for each study participant based on the percentage of correctly answered questions, taking into account the level of difficulty of each question and the total number of questions answered by the study participants; multiple choice questions were awarded 1 points when answered correctly, where in open question women received up to 3 points for correct statements. Participants that answered less than 50% of the questions were excluded from the analysis.

Mixed model multivariate regression analysis, controlling for dependency per individual and per household, was performed to investigate the association between the education campaign and level of knowledge of malaria in the community, adjusting for socio-economic and demographic confounders.

Answers given in the questionnaires were recoded binomially; "right" or "wrong". Subsequently, the McNemar's test was performed in order to assess improvement in level of knowledge on each topic/question, accounted for dependency.

All analyses were performed in Stata version 11.

## Results

### Respondent characteristics

In total 2112 eligible women (residents and visitors) were asked to participate in the study. Of these women, 157 (7.4%) refused participation or quit the interview before completion. After the peer-education campaign this process was repeated; 2040 women were approached for participation in the post-intervention assessment, of which 174 refused participation. In total, 1955 women of reproductive age living or visiting the study area participated in the pre-education study and 1866 women in the post-study. From 1105 women, pre- and post education answers could be matched for longitudinal analysis. In the remaining households two different family members answered the questions before and after the intervention, and were excluded from the longitudinal analysis.

Just over 43% of the participants are living in an urban area. Most of them (63%) are married. More then half of the study participants (58.3%) received secondary education or higher and 12.7% never went to school. Of all participants 62.1% had been pregnant before. All the baseline characteristics of the study participants can be found in Table 1. Due to misreporting or data entry mistakes, for some participants differences in pre and post measurements of demographic data were observed. In that situation, the post education records were used for the analysis.

**Table 1:** Demographic characteristics of interviewed women

		N	%
Living Area			
	*Urban*	477	43.1
	*Rural*	624	56.4
	*Unknown*	5	0.5

Age			
	*Median*	28	
	*Range*	15-49	

Marital Status			
	*Married*	700	63.3
	*Single*	373	33.7
	*Separated*	13	1.2
	*Divorced*	7	0.6
	*Unknown*	13	1.2

Level of education			
	*None*	140	12.7
	*Primary*	320	28.9
	*Secondary*	592	53.5
	*Higher*	48	4.3
	*Unknown*	6	0.5

Pregnancy Status			
	*Pregnant at intake*	66	6.0
	*Ever been pregnant*	687	62.1
*Of which:*			
	*In the past 5 years*	341	49.6
	*Longer than 5 years ago*	309	45.0
	*Unknown*	37	0.4
	*Never been pregnant*	415	37.5
	*Unknown*	4	0.4

### Knowledge on malaria

In the pre-assessment women on average answered 64.8% of the question correctly. The peer education campaign had a significant impact in raising the level of knowledge among the women; after the campaign the respondents answered on average 73.8% of the questions correctly. Stratified analysis on pre and post assessment scores for malaria in general (68.8 & 72.9%) and malaria in pregnancy (61.7 & 76.3%) showed also significant increase. Uptake of bed nets was reported to be low: 11.6%

Table 2 summarizes the percentages of correctly answered questions during pre and post intervention studies. The knowledge of the study participants increased significantly on all topics, except knowledge on transmission of malaria and malaria symptoms.

**Table 2:** Overview of respondents answers to questionnaire items before and after the intervention

Questionnaire items	Pre intervention		Post intervention		Difference	P-value (McNemar's)
	N	%	N	%	%	
Malaria characteristics in general						
*Correct*	683	61.81	748	67.88		
*Incorrect*	422	38.19	354	32.12	5.9	0.0038

Is malaria a problem in the area?						
*Correct*	982	95.71	1005	98.63		
*Incorrect*	44	4.29	14	1.37	3.2	< 0.0001

How is malaria transmitted?						
*Correct*	993	90.93	1012	93.01		
*Incorrect*	99	9.07	76	6.99	2.0	0.0882

What can be done to prevent malaria?						
*Correct*	200	18.21	365	32.48		
*Partially correct*	780	71.04	699	63.78		
*Incorrect*	118	10.75	41	3.74	(pooled) 7.1	< 0.0001

Symptoms of malaria						
*Correct*	626	56.65	569	51.63		
*Incorrect*	479	43.35	533	48.37	- 5.1	0.0120

Can malaria occur during pregnancy?						
*Correct*	898	81.93	1022	96.96		
*Incorrect*	198	18.07	32	3.04	13.2	< 0.0001

Susceptibility during pregnancy?						
*Correct*	768	73.07	895	85.98		
*Incorrect*	283	26.93	146	14.02	11.3	< 0.0001

Can malaria give problems in pregnancy?						
*Correct*	820	77.14	990	96.40		
*Incorrect*	243	22.86	37	3.60	17.6	< 0.0001

Can malaria give problems for the foetus?						
*Correct*	765	82.79	973	97.30		
*Incorrect*	159	17.21	27	2.70	15.4	< 0.0001

What problems can malaria give?						
*Correct*	96	9.04	263	23.91		
*Partially correct*	780	73.45	721	65.55	(pooled) 7.2	< 0.0001
*Incorrect*	186	17.51	116	10.55		

How can malaria be prevented during pregnancy?						
*Correct*	26	2.47	73	6.80		
*Partially correct*	782	74.33	885	82.48	12.1	< 0.0001
*Incorrect*	244	23.19	115	10.72		

Is correct use of IPTp (Fansidar) named?						
*Correct*	69	6.23	214	19.35		
*Incorrect*	98	8.86	41	3.71	28.6	0.0005
*No answer*	939	84.90	851	76.94	*Excluded*	

What medications for malaria can be used during pregnancy?						
*Correct*	520	50.05	686	64.78	14.5	< 0.0001
*Incorrect*	519	49.95	373	35.22		

Almost all (> 90%) women had heard about malaria and the way it is transmitted. Before the intervention, 73% of the respondents knew that pregnant women were more susceptible to contracting malaria and that malaria could cause problems during pregnancy; after the intervention there was a significant increase to > 90% of the respondents. Knowledge on preventive measures also increased significantly; while around 50% of the participants were aware that bed nets could prevent malaria before the intervention, this increased to around 80% after the intervention. Level of knowledge on use of IPTp in the prevention of malaria during pregnancy remained very low: 6% of respondents could correctly name the drug and how to use it versus 19% after the intervention.

Table 3 shows the results of the multilevel mixed-effects linear regression on the 1105 women who have been interviewed before and after the intervention (controlling for dependency). It shows that the adjusted increase in knowledge "score" due to the peer education was 9.36 points: after the peer education campaign women answered the questions on average the almost 10% better than before the peer education. Other significant factors that increased the knowledge score were "ever been pregnant" and "history of malaria infection in the last three months" Women that had ever been pregnant scored on average better (6.69%) than women that had not been pregnant before and women that had suffered from malaria in the last three months scored 5.82% better than women that never had malaria. Women living in an urban area were on average scoring slightly better than women in rural areas (70.7% versus 68.2%).

**Table 3:** Multilevel mixed-effects linear regression analysis of effect of peer education and other personal factors on knowledge score of women of reproductive age (N = 1105 interviewed before and after the intervention)

Variable	Average score	Crude Coefficient	Adjusted Coefficient	P-value	95% Conf. Interval	
					**Lower limit**	**Upper limit**

Peer education:						
*Pre peer education*	64.8		-			
*Post peer education*	73.8	9.0	9.36	< 0.001	8.01	10.70

Age *(per year increase)*		0.2	0.002	0.975	- 0.11	0.11

Living Area						
*Urban*	70.7		-			
*Rural*	68.2	-2.5	-1.59	0.057	- 3.23	0.05

Level of education						
*None*	69.6		-			
*Primary*	69.6	0	- 0.24	0.855	-2.85	2.36
*Secondary*	69.2	-0.4	1.28	0.354	-1.42	3.98
*Higher*	68.7	0.1	1.56	0.492	-2.89	6.00

Pregnancy Status						
*Ever been pregnant*	71.5		-			
*Never been pregnant*	65.7	-5.8	-6.69	< 0.001	-8.99	-4.39

History of malaria						
*Had malaria in the last three months*	72.4		-			
*Never had malaria*	66.1	-6.3	-5.82	< 0.001	-7.66	-4.30

### Preventive practices during previous pregnancies and current pregnancies

Table 4 summarizes the responses on preventive practice questions from women that have been pregnant before and/or are currently pregnant. Answers before and after the education campaigns were not compared, due to the limited number of pregnant participants in the post-questionnaire round.

**Table 4:** Preventive practices among pregnant women who were pregnant during the intervention or have been pregnant before.

	N	%
Visiting antenatal care provider (multiple answers possible) (**N_tot _= 645)**		
Doctor	388	60.2
Nurse/midwife	478	74.1
CHW	36	5.6
Traditional birth attendant	26	4.0
Other care provider	4	06
***Total: Any care provider***	***630***	***97.7***
***No***	***15***	***2.3***

Number of participants sleeping under net during pregnancy (**N_tot _= 636)**		
Yes	74	11.6
No	562	88.4

Preventative medication during pregnancy (multiple answers possible) (**N_tot _= 624)**		
Yes, from the hospital	446	71.5
Yes, from the pharmacy	157	25.2
Yes, from a traditional birth attendant	25	4.0
Yes, elsewhere	5	0.8
***Total: Yes, from any source***	***481***	***77.1***
***No***	***143***	***22.9***

Medicines used to prevent malaria		
SP/Fansidar^®^	65	11.8
Chloroquine	160	29.0
Pyrimethamine (Daraprim^®^)	102	21.8
Other (non malaria drugs)	31	5.6
Can't remember	192	34.9

Remarkable is the high percentage of women that do already attend an antenatal health care provider. Also the uptake of anti-malarial drugs was high including prophylactic treatment. However, over half of the interviewees used anti-malarial drugs that are not recommended for malaria prevention during pregnancy or appropriate for IPTp. Of the women that did not use any drugs to prevent malaria, 40% reported that they did not take drugs because they were not given to them during their pregnancy. Other answers on the question why they did not use preventive drugs were: "drugs were given but was afraid to use them" or "drugs were given but I do not know how to use them". A third of the women could not remember what drug was given to prevent malaria. Of the women that could remember what was given only 10% reported that they were given the recommended treatment with SP.

Bed net use seems to be very low; 97% of the respondents that did not sleep under a bed net reported that they did not have one at home. Other remarks mentioned for not using a bed net were: "I have a bed net but do not know how to use it, "I have a bed net but do not like to sleep under it" and "I want to buy a net but I cannot find one".

Multilevel logistic regression revealed that living in an urban area was significantly associated with using a bed net during pregnancy (OR = 2.07; P = 0.02; 95%CI [1.12-3.81]. Other demographic, disease and knowledge factors were not found to be associated with bed net use.

### Knowledge and preventive practice in current pregnancies

There were 66 women participating in the study that were pregnant at intake. During the revisit after the peer education campaign 17 of them were still pregnant. The other 49 had delivered within the period of the study. The average knowledge score after peer education (74.2 points) was higher than before peer education (69.0 points). The one-sided P-value of the two sample paired T-test showed that this difference was significant (P = 0.0312). Due to non-reporting the pre-and post questionnaires, preventative action among pregnant women could not be compared.

## Discussion

This study found that peer education could be seen as an effective tool to increase the knowledge of young women about malaria. Multilevel mixed-effects linear regression revealed that besides peer education, living area (urban vs. rural), pregnancy status and history of malaria in the last three months had influence on the knowledge score of the women.

The use of bednets and IPTp-sp was found to be very low in the population. Similar findings have been reported among pregnant women attending antenatal clinics in some health facilities in the state [9,16]. Although this study did not specifically address the reasons why preventive measure uptake was low, this could possibly be explained by structural barriers. A majority of the respondents mentioned that they did not have access to for example bed nets or did not know how to use them. Others mentioned that they did not know how to use the anti-malarial medication or that they were not available at the clinic. This shows that there may also be a need to pay extra attention to the education of health care providers and bed net distributors to enhance their knowledge and skills on bed net use, which will subsequently be transferred to the end users.

Some studies have shown that uptake of preventive measures is especially difficult in adolescent girls [19]. Although the response rate of the study population of pregnant women and the time span of the study was too small to see if there is a significant difference in uptake of preventive measures in this age group the study did show that the level of knowledge was also significantly related to age. The older the women the more the women knew about the preventive measures.

Some limitations exist in this study. Our inability to completely match the same respondent at both pre- and post- intervention survey results was a major limitation. This might be due partly to the study design that made a woman eligible if she slept in the household at least the night before the questionnaire was administered, which means that visitors would also have been included in the study. Another explanation may be possible migration of women out of the study area after pre- but before post-intervention survey.

A sub-analysis between those who were interviewed twice and all women who were enrolled in the pre-questionnaire round did not show a great difference between the responses and characteristics of the groups (data not shown). However, it should be noted that interpreting data from all the interviewed women (including visitors) should be done with caution as visitors may come from a different information background and thus using these results may give a misinterpretation of the situation in the specific population under study.

Another limitation was the limited number of women that were pregnant or became pregnant during the study period. This limits the possibilities for sub-analysis of preventive practice at this level. A small proportion of women had been pregnant recently (over 50% had been pregnant more than 5 years ago) which may affect the answers given on preventive practices used during their pregnancy. Recall bias or changed information over time may have affected the results. Future studies may specifically focus on recruiting an additional group of women based on their pregnancy status during the intervention.

This study was based solely on qualitative and quantitative data from structured questionnaires. Although the study finds that peer education is a very effective tool to raise awareness in the community, there was limited data available to asses if there was a direct effect on the practice of women. Further studies should thus take into account a way to analyze the true uptake. In addition, true malaria incidence data would also be helpful because even if this study showed an increased effect of preventive measures in the questionnaire it does not necessarily reflect a reduction of malaria infections. A study combining these factors would thus be beneficial in order to measure the true effect of an education campaign.

## Conclusion

Prior to planning community-based health education interventions, it is important to conduct properly designed surveys to assess levels of knowledge already present in the communities. This study found that the knowledge of malaria in general was high but that of malaria in pregnancy and its preventive measures was low. In addition, uptake of antimalarials to prevent malaria in pregnancy was also found to be low.

The peer education campaign had a significant impact in raising the knowledge of women of child bearing age on malaria in pregnancy and its preventive measures. There were, however, limited data to assess whether this increased knowledge also translates in increased uptake of the preventive practices. Future studies and health interventions should thus consider other factors influencing preventive practices such as knowledge, structural barriers and lack of prevention tools (bed nets, nearby health facilities) and should address these issues as well.

## Competing interests

The authors declare that they have no competing interests.

## Authors' contributions

PM: conception and design of the study, interpretation of the data and drafting of the manuscript. PS: Data analysis and interpretation of the data. Significant contribution to the writing and critical reading of the manuscript. HA: data analysis and interpretation of the data. EE: Study design and its execution in Nigeria, data collection and study monitoring and critical reading of the manuscript.

All authors read and approved the final manuscript.

## Pre-publication history

The pre-publication history for this paper can be accessed here:

http://www.biomedcentral.com/1471-2458/11/610/prepub
